# Congenital onychodysplasia of the index fingers (Iso-Kikuchi syndrome): A systematic review of the clinical spectrum

**DOI:** 10.1016/j.jdin.2026.06.024

**Published:** 2026-07-28

**Authors:** Yeanna Moon, Ji Su Lee, Bo Ri Kim, Adam I. Rubin, Je-Ho Mun

**Affiliations:** a University of Missouri – Kansas City School of Medicine, Kansas City, Missouri; b Department of Dermatology, Seoul National University College of Medicine, Seoul, Korea; c Department of Dermatology, Seoul Metropolitan Government National University (SMG-SNU) Boramae Medical Center, Seoul, Korea; d Department of Dermatology, Seoul National University Bundang Hospital, Seongnam, Korea; e Ronald O. Perelman Department of Dermatology, NYU Grossman School of Medicine, New York, New York; f Department of Dermatology, Seoul National University Hospital, Seoul, Korea

**Keywords:** congenital abnormalities, congenital onychodysplasia, Iso-Kikuchi syndrome, nails, nail diseases, nail dysplasia

*To the Editor:* Congenital onychodysplasia of the index fingers (COIF), also known as Iso-Kikuchi syndrome, is a rare nail disorder predominantly affecting the index fingers, with occasional involvement of other digits. Initially described by Iso in 1969, the condition was later termed COIF by Kikuchi.^1^ Nail findings in COIF can include polyonychia, micronychia, and anonychia. The diagnostic criteria, revised by Baran and Stroud^2^ in 1984, include congenital occurrence, unilateral or bilateral index finger involvement, variability in nail appearance, hereditary predisposition, and associated bone anomalies. The pathogenesis remains unclear but has been hypothesized to involve localized intrauterine mechanical or vascular disturbances affecting nail unit development.^3^ Although initially thought to involve only the index fingers, reports have documented the involvement of other digits.^3^^,^^4^ Given its rarity, we conducted a systematic review to summarize existing literature and characterize its clinical spectrum.

This systematic review was conducted in accordance with the Preferred Reporting Items for Systematic Reviews and Meta-Analyses guidelines. We searched PubMed, Embase, and Cochrane databases using the terms “congenital onychodysplasia of the index finger(s),” “congenital onychodysplasia,” and “Iso-Kikuchi syndrome” on November 10, 2024, and updated the search on June 8, 2026. Abstracts were screened, followed by a full-text review by three reviewers (YM, JSL, and BRK). Studies were included if they reported COIF or Iso-Kikuchi syndrome with detailed information on patient demographics, affected digits, nail abnormalities, radiologic findings, family history, or treatment outcomes. Both classic cases involving the index fingers and atypical cases involving other digits or toes were included if described within the COIF/Iso-Kikuchi spectrum. Studies unrelated to COIF and those not published in English were excluded.

After screening, 40 studies comprising 82 patients met the inclusion criteria (Supplementary Table I, available via Mendeley at https://data.mendeley.com/datasets/42bnnm6w7g/1; Supplementary Figure 1, available via Mendeley at https://data.mendeley.com/datasets/42bnnm6w7g/1; and Supplementary References, available via Mendeley at https://data.mendeley.com/datasets/42bnnm6w7g/1). The mean age at diagnosis was 17.1 years (SD, 19.1; range, 0.08-85 years; Table I). The sex distribution was nearly equal, with 52.4% and 47.6% of the cases occurring in females and males, respectively. Single-digit involvement was observed in 40.2% of the cases, whereas multiple-digit involvement was observed in 59.8% of the cases. COIF mostly affected the fingers (92.7%); 3.7% of the cases involved both the fingers and toes, and 3.7% involved only the toes. Among the affected digits, both index fingers, the left index finger, and the right index finger were primarily involved in 48.8%, 28.0%, and 6.1% of the cases, respectively. Involvement of other digits, including the third through fifth fingers and various toes, was reported in 17.1% of cases. Radiologic assessments were reported in 75.6% of cases. Imaging findings included hypoplasia and Y-shaped bifurcation of the distal phalanx (40.3%), Y-shaped bifurcation of the distal phalanx (24.2%), hypoplasia of the distal phalanx (16.1%), and other anomalies (Table II).

**Table I:** Patient demographics and clinical characteristics of congenital onychodysplasia of the index fingers (*n* = 82)

Characteristic (*n* = 82)	Value
Mean age, y (SD)	17.1 (19.1)
Age range, y	0.08-85
Sex, *n* (%)	
Female	43 (52.4)
Male	39 (47.6)
Digit involvement	
Single-digit involvement	33 (40.2)
Multiple-digit involvement	49 (59.8)
Fingers and/or toe involvement, *n* (%)	
Fingernail involvement only	76 (92.7)
Toenail involvement only	3 (3.7)
Both fingernail and toenail Involvement	3 (3.7)
Affected digits, *n* (%)	
Both indexes	40 (48.8)
Left index	23 (28.0)
Right index	5 (6.1)
Both indexes and right third finger	3 (3.7)
Both indexes and left third finger	1 (1.2)
Both indexes and both first fingers	1 (1.2)
Both third fingers	1 (1.2)
Left third finger	1 (1.2)
Left fourth finger	1 (1.2)
Both indexes and left second toe	1 (1.2)
Both indexes, left third finger, and left first toe	1 (1.2)
Both indexes and right first through fourth toes	1 (1.2)
Unilateral second toes	2 (2.4)
Right second toe	1 (1.2)

**Table II:** Clinical features, radiologic findings, and treatment of congenital onychodysplasia of the index fingers

Characteristic (*n* = 82)	Value
Nail findings, *n* (%)∗	
Micronychia	60 (73.2)
Polyonychia	31 (37.8)
Anonychia	18 (22.0)
Hemionychogryposis	4 (4.9)
Absent lunulae	6 (7.3)
Blue lunulae	2 (2.4)
Leukonychia	1 (1.2)
Imaging performed, *n* (%)	
Radiography	62 (75.6)
Radiologic findings (*n =* 62), *n* (%)†	
Hypoplasia and Y-shaped bifurcation of distal phalanx	25 (40.3)
Y-shaped bifurcation of distal phalanx	15 (24.2)
Hypoplasia of the distal phalanx	10 (16.1)
No underlying bony abnormality	9 (14.5)
Brachydactyly‡	2 (3.2)
Complete osseous fusion of phalanx	1 (1.6)
Perinatal drug exposures, *n* (%)	
Progestins	2 (2.4)
Antiemesis	1 (1.2)
Insulin	1 (1.2)
Valium (diazepam)	1 (1.2)
Abortifacient	1 (1.2)
Mebendazole	1 (1.2)
Hep B immune globulin and hepatitis B vaccine	1 (1.2)
Hereditary or familial features, *n* (%)	
Autosomal dominant inheritance	11 (13.4)
Familial nail dysplasia of unknown etiology	7 (8.5)
Born from consanguineous marriage	2 (2.4)
Treatment, *n* (%)	
Treatment was not performed	81 (98.8)
Treatment was performed	1 (1.2)

∗ Patients may have multiple nail findings.

† Percentages are calculated based on 62 patients who underwent imaging.

‡ One case was specifically designated as brachymesophalangia, a subtype of brachydactyly affecting the middle phalanx.

While most cases were idiopathic, autosomal dominant inheritance (13.4%), family history of nail dysplasia (8.5%), maternal drug exposure (9.8%), and birth from consanguineous marriage (2.4%) were noted (Table II). Management was generally conservative in asymptomatic cases because there was no functional impairment. One case described the treatment of a pincer nail deformity in COIF using a shape-memory alloy nail device.^5^

Limitations of this review include the retrospective design, small sample size, exclusion of non-English studies, potential publication bias, and heterogeneity in reporting. In addition, several variables—such as family history, prenatal/perinatal history, and treatment data—were inconsistently reported across studies, precluding reliable denominator-based analyses; thus, absence of reporting should not be interpreted as absence of findings, introducing potential reporting and selection bias.

To our knowledge, this is the first systematic review of COIF. Although COIF predominantly affects the index digits, involvement of other digits has been documented, suggesting a broader clinical spectrum. Further studies are needed to better understand the disease spectrum and pathomechanism.

## Conflicts of interest

None disclosed.
